# Impact of high-risk pregnancy on the work routine of pregnant women in Catalão, Brazil

**DOI:** 10.47626/1679-4435-2025-1394

**Published:** 2025-11-04

**Authors:** Rebeca de Paula Milward Azevedo, Caio Alves Lemos, Gizelda Vasconcelos Vieira de Alcântara, Thaynara Araujo de Assis

**Affiliations:** 1 Institute of Biotechnology, Universidade Federal de Catalão, Catalão, GO, Brazil

**Keywords:** high-risk pregnancy, workforce, employment relationship., gravidez de alto risco, mercado de trabalho, relações trabalhistas.

## Abstract

**Introduction:**

Pregnancy is a period in a woman’s life characterized by expectations, desires, and profound physical, emotional, and social changes. These transformations require multiple adjustments, including in the professional sphere, as pregnancy may limit women’s ability to perform their usual work activities and affect their employment relationship.

**Objectives:**

To analyze the changes and impacts on the professional lives of high-risk pregnant women in the city of Catalão, state of Goiás, Brazil.

**Methods:**

This was a cross-sectional analytical study conducted at the obstetrics service of the Women’s Integrated Center in Catalão, Brazil.

**Results:**

Of the 123 high-risk pregnant women interviewed, 7 (5.7%) were between 13 and 17 years old, 54 (43.9%) were between 18 and 29 years old, 45 (36.6%) were between 30 and 39 years old, and 17 (13.8%) were aged 40 years or older. A total of 82 (66.7%) women were employed outside the home. Regarding the changes experienced, 84 (68.3%) reported that pregnancy led to changes in their daily routine, 32 (38.1%) reported changes in their work routine, and 64 (76.2%) stated that these changes caused emotional impacts.

**Conclusions:**

Work and pregnancy directly influence women’s lives, with professional activity often serving as a primary means of family support. However, in high-risk pregnancies, psychological or physical factors frequently require work leave. Moreover, as work is a source of personal fulfillment for many women, such imposed changes can result in significant financial, emotional, and social impacts.

## Abstract (pt)

**Introdução:**

O gestar, na vida da mulher, é um momento que gera expectativas e anseios, além de mudanças físicas, emocionais e sociais. Tais mudanças implicam adaptações necessárias, inclusive no contexto profissional, uma vez que a gravidez provoca alterações que podem limitar a mulher e sua relação profissional.

**Objetivos:**

Analisar as mudanças e os impactos na vida profissional de gestantes de alto risco do município de Catalão, estado de Goiás.

**Métodos:**

Trata-se de um estudo analítico transversal, realizado no serviço de obstetrícia do Centro Integrado da Mulher, no município de Catalão, estado de Goiás.

**Resultados:**

Do total de 123 gestantes entrevistadas, 7 (5,7%) tinham entre 13 e 17 anos, 54 (43,9%), entre 18 e 29 anos, 45 (36,6%), entre 30 e 39 anos e 17 (13,8%) tinham 40 anos ou mais; 82 (66,7%) trabalhavam fora de casa. Em relação às mudanças vivenciadas, 84 (68,3%) afirmaram que a gestação implicou alterações na rotina da vida, 32 (38,1%) relataram mudanças na rotina do trabalho e 64 (76,2%) disseram que essas mudanças causaram impactos emocionais.

**Conclusões:**

O trabalho e a gestação influenciam diretamente a vida da mulher, estando a função profissional definida como um meio de sustento familiar. Contudo, nas gestações de alto risco, seja por fatores psicológicos ou físicos, as gestantes frequentemente necessitam ser afastadas de seus cargos. Além disso, para muitas mulheres, o trabalho também está relacionado à satisfação pessoal. Assim, as mudanças impostas geram impactos financeiros, emocionais e sociais.

## INTRODUCTION

Pregnancy is a period in a woman’s life marked by multiple physical, social, and emotional transformations that occur simultaneously within a remarkably brief time frame.^1^ Although physiological in nature, these changes influence the level of care required during the prenatal, intrapartum, and postpartum phases, as determined by the degree of gestational risk attributed to the pregnant woman. When such changes do not occur favorably, women are classified as having intermediateor high-risk pregnancies.^2^

Situations experienced by women during pregnancy, related to adverse socioeconomic conditions, preexisting diseases, poor obstetric history, complications arising during the current pregnancy, or even fetal abnormalities, may place them in a condition of greater vulnerability and gestational risk. In such cases, the pregnancy is considered high-risk.^3^ High-risk pregnancies generally trigger behavioral, affective, and emotional issues, which can increase stress levels and reduce self-esteem, in addition to interfering with the fetus’ neurodevelopment.^4^

Furthermore, beyond physical changes, mood swings are also observed among these women. Self-esteem, though little addressed, may be a significant aspect in assessing preparedness for motherhood. Women with a negative self-image and low self-assessed competence may view themselves as incapable of fulfilling the maternal role satisfactorily, especially if they are younger and have limited social support.^5^

Maternal stress, social support, and self-esteem are important factors to be considered in any pregnancy, as they directly influence fetal development, the satisfactory progression of pregnancy, and the construction of the maternal role. When applied to high-risk pregnancies - during which women experience heightened fear, increased dependency on others, and a greater sense of incapacity -, these factors become even more significant.^6^

All these aspects combine and result in several changes experienced by women during pregnancy and the puerperium. Moreover, it is important to emphasize the role of work, understood through the lenses of sociology and psychology, as an essential element in the construction of individual identity and as a means of social ascension and integration.^7^

Therefore, this study aimed to investigate the changes and professional impacts experienced by high-risk pregnant women in the municipality of Catalão, in the state of Goiás, Brazil.

## METHODS

### STUDY DESIGN

This was an analytical cross-sectional study conducted at the obstetrics service of the Women’s Integrated Center (*Centro Integrado da Mulher*) in the municipality of Catalão, Brazil. After intake and gestational risk stratification by the primary healthcare team at the healthcare units, pregnant women who did not meet the criteria for an uncomplicated pregnancy were referred to the municipality’s high-risk prenatal service.

The assessment tools used were a sociodemographic profile questionnaire, developed by the researchers, and the Prenatal Psychosocial Profile (PPP-VP), Portuguese version.

### ETHICAL ASPECTS

This study was approved by the Research Ethics Committee of the Federal University of Catalão (CAAE No. 65527722.2.3001.0164) and by the Research Ethics Committee of PUC Goiás (CAAE No. 65527722.2.0000.0037). Participants were properly informed about the study objectives, potential risks, confidentiality of information, and assurance of privacy during the interview. After clarifying any questions, and upon reading, agreeing to, and signing the Informed Consent Form (for pregnant women aged 18 years or older) or the Informed Assent Form (for pregnant women under 18 years of age and their legal guardians), data collection was initiated.

## RESULTS

Of the 130 pregnant women invited to participate in the study, seven declined, resulting in a final sample of 123 high-risk pregnant women who met the inclusion criteria. Table 1 presents the results related to their sociodemographic profile and the changes that pregnancy brought to their lives.

**Table 1:** Sociodemographic profile, changes in daily routine and work, and emotional impact among high-risk pregnant women treated in the public health system of a municipality in the state of Goiás, Brazil

	n	%
Age group, years		
13-17	7	5.7
18-29	54	43.9
30-39	45	36.6
≥ 40	17	13.8
Employed outside the home		
No	41	33.3
Yes	82	66.7
Changes in daily routine		
No	39	31.7
Yes	84	68.3
Changes in work routine		
No	95	77,2
Yes	32	38.1
Emotional impact		
No	59	47,9
Yes	64	76.2

% = relative frequency; n = absolute frequency.

Regarding age distribution, seven women (5.7%) were between 13 and 17 years old, 54 (43.9%) were between 18 and 29 years old, 45 (36.6%) were between 30 and 39 years old, and 17 (13.8%) were 40 years old or older. Of all participants, 82 (66.7%) were employed outside the home. Concerning the changes experienced, 84 (68.3%) reported that pregnancy brought alterations to their daily routine, 32 (38.1%) reported changes in their work routine, and 64 (76.2%) stated that these changes caused emotional impacts.

## DISCUSSION

Pregnancy is a condition characterized by physical, hormonal, social, family, and psychological changes. Many women, depending on their personal history, lifestyle habits, and available support systems, exhibit significant vulnerability to mental health issues.^8^ The psychological dimensions of women experiencing high-risk pregnancies are even more complex, as they demand an intense process of emotional regulation. Maternal experience becomes more challenging due to the emotional vulnerability inherent to this period, the increased risks involved, and the wide range of emotions associated with their clinical condition^9^ - a complexity of feelings that reverberates across different areas of their lives, including the professional sphere.

With the increasing participation of women in the workforce, they have come to play an essential role in supporting their families, often assuming the role of main provider and being forced to balance their professional careers with motherhood. However, in some cases of high-risk pregnancies, whether due to psychological or physiological factors, women may need to step away from work because they are unable to perform their usual duties. This situation represents a significant hardship for workers without a formal employment relationship, such as the self-employed, freelancers, or independent contractors, who may lack legal protection against dismissal during pregnancy. Consequently, this can lead to job loss and additional financial difficulties.

Another important aspect is the emotional impact experienced by these women, particularly in the relationship between work and pregnancy. The fear of dismissal, the distress of returning to work and not resuming the previously held position, the anxiety about being separated from the baby upon returning to work, and the fear that high-risk pregnancy conditions might worsen due to work activities all contribute to making this period one of intense internal conflict. For women at this stage, “motherhood and work are imaginatively experienced as opposing competitors in the pursuit of phallic fulfillment, in which increasing investment in one necessarily implies proportional disinvestment in the other.”^10^

Moreover, work and pregnancy also trigger significant changes in family life. With new configurations in family structure, women have ceased to fulfill exclusively the roles of wife and mother and have taken on professional activities in the private or public sectors, thus becoming family providers as well. This dynamic represents a reorganization of social roles, which occurs bilaterally, as men also undergo substantial changes. Consequently, transformations in the roles of mother and father within the family become evident, reflecting broader shifts in the understanding of motherhood.^10^

Another change observed in contemporary society is the postponement of motherhood, which is associated with the increasing participation of women in the workforce - which, in the past, was mostly composed of men. This reality is particularly evident among women who prioritize and value their professional careers, resulting in a conflict between two central priorities of adult life: becoming a mother or establishing one’s career. As women experience their reproductive and productive phases simultaneously - both of which demand significant dedication and commitment -, this dilemma becomes even more evident.^11^

The social, cognitive, and emotional benefits provided by work play an important role in motivating working mothers to face the inherent challenges of balancing the multiple roles that require substantial dedication from them.^12^ For many women, work extends beyond financial necessity; it also offers satisfaction and a sense of personal and social fulfillment. In this context, when they need to leave the workforce during pregnancy, they often perceive that they are no longer the same person they were before and that their role in the world has changed - feelings that negatively affect mental health, especially among those already experiencing greater emotional vulnerability.

## CONCLUSIONS

High-risk pregnancies are always challenging for women, not only in physical and emotional terms but also in the social and professional spheres. The results of this study demonstrate that high-risk pregnant women experience significant changes in their personal and work lives, which often impact their emotional health and self-esteem. The need to balance motherhood with job demands is a source of stress and internal conflict, especially among those in more vulnerable socioeconomic conditions or without legal employment protection.

Furthermore, changes in social roles - marked by the increasing participation of women in the workforce and the reorganization of family structures - underscore the importance of public policies and initiatives that ensure emotional, social, and financial support for pregnant women. Such measures are essential to mitigate the negative impacts of high-risk pregnancies, promoting not only maternal well-being but also the healthy development of the fetus and the stability of families.

Despite the study’s limitations, such as the subjectivity of the topic and the difficulty of comprehension among participants with lower educational levels, this research contributes to a broader understanding of the challenges faced by high-risk pregnant women in the municipality of Catalão, Brazil. It highlights the need for targeted interventions that consider the specific circumstances of this group, as well as their relationship with work.

## References

[1] Brasil, Ministério da Saúde (2012). Gestação de alto risco: manual técnico
(Série A. Normas e Manuais Técnicos).

[2] Alves AC, Cecatti JG, Souza RT. (2021). Resilience and stress during pregnancy: a comprehensive
multidimensional approach in maternal and perinatal health. ScientificWorldJournal.

[3] Gadelha IP, Souza RT, Cecatti JG. (2020). Determinantes sociais da saúde de gestantes acompanhadas
no pré-natal de alto risco. Rev Rene.

[4] Nolvi S, Merz EC, Kataja EL, Parsons CE. (2023). Prenatal stress and the developing brain: postnatal environments
promoting resilience. Biol Psychiatry.

[5] Kucharska J. (2018). Validation of the Polish version of the Posttraumatic Cognitions
Inventory. Curr Psychol.

[6] Yu X, Liu Y, Huang Y, Zeng T. (2022). The effect of nonpharmacological interventions on the mental
health of high-risk pregnant women: a systematic review. Complement Ther Med.

[7] Blanch-Ribas JM. (2003). Teoría de las relaciones laborales: fundamentos.

[8] Morais AODS, Simões VMF, Rodrigues LS, Batista RFL, Lamy ZC, Carvalho CA (2017). Sintomas depressivos e de ansiedade maternos e prejuízos
na relação mãe/filho em uma coorte pré-natal:
uma abordagem com modelagem de equações
estruturais. Cad. Saude Publica.

[9] Caldas DB, Silva ALR, Böing E, Crepaldi MA, Custódio ZAO. (2013). Atendimento psicológico no pré-natal de alto risco:
a construção de um serviço. Psicol Hosp (São Paulo).

[10] Beltrame GR, Donelli TMS. (2012). Maternidade e carreira: desafios frente à
conciliação de papéis. Aletheia.

[11] Barbosa PZ, Rocha-Coutinho ML. (2007). Maternidade: novas possibilidades, antigas
visões. Psicol Clin.

[12] Toledo JS, Martins JT, Gonçalves J. (2023). Percepções de mulheres sobre gestação
e os sentidos do trabalho. Cad Psicol Soc Trab.

